# Strategies Based on Nitride Materials Chemistry to Stabilize Li Metal Anode

**DOI:** 10.1002/advs.201600517

**Published:** 2017-03-03

**Authors:** Yizhou Zhu, Xingfeng He, Yifei Mo

**Affiliations:** ^1^ Department of Materials Science and Engineering University of Maryland College Park MD 20742 USA; ^2^ University of Maryland Energy Research Center University of Maryland College Park MD 20742 USA

**Keywords:** first‐principles calculations, lithium‐ion batteries, lithium metal anodes, solid‐electrolyte‐interphase, all‐solid‐state batteries

## Abstract

Lithium metal battery is a promising candidate for high‐energy‐density energy storage. Unfortunately, the strongly reducing nature of lithium metal has been an outstanding challenge causing poor stability and low coulombic efficiency in lithium batteries. For decades, there are significant research efforts to stabilize lithium metal anode. However, such efforts are greatly impeded by the lack of knowledge about lithium‐stable materials chemistry. So far, only a few materials are known to be stable against Li metal. To resolve this outstanding challenge, lithium‐stable materials have been uncovered out of chemistry across the periodic table using first‐principles calculations based on large materials database. It is found that most oxides, sulfides, and halides, commonly studied as protection materials, are reduced by lithium metal due to the reduction of metal cations. It is discovered that nitride anion chemistry exhibits unique stability against Li metal, which is either thermodynamically intrinsic or a result of stable passivation. The results here establish essential guidelines for selecting, designing, and discovering materials for lithium metal protection, and propose multiple novel strategies of using nitride materials and high nitrogen doping to form stable solid‐electrolyte‐interphase for lithium metal anode, paving the way for high‐energy rechargeable lithium batteries.

## 1 Introduction

Li metal has been long desired as the anode material with the highest theoretical specific capacity and the lowest standard potential to significantly increase the energy density in rechargeable Li‐ion battery.^1^, ^2^ Enabling Li metal anode has been regarded as the “Holy Grail,”^3^, ^4^ but confronts many challenges. The undesired growth of Li dendrite during cell cycling causes short circuiting in the cell, leading to catastrophic cell failure and safety issues. In addition, Li metal is strongly reducing, and is not compatible with most electrolytes.^5^, ^6^, ^7^ The lack of long‐term stability between electrolyte and Li metal anode results in low coulombic efficiency, capacity fading during cycling, and cell failure.^6^, ^7^, ^8^ To protect electrolytes against Li metal and to stabilize Li metal anode, electrolytes and additives are developed to form stable solid‐electrolyte‐interphase (SEI) layer on Li metal.^8^ Forming such SEI layers has led to the success of graphite anode in commercial Li‐ion batteries.^5^, ^6^, ^9^, ^10^ Besides spontaneous formation of SEI, applying Li‐stable protection materials as artificial SEI on Li metal is also demonstrated to significantly improve the cyclability and coulombic efficiency of the cells with Li metal anode.^11^, ^12^, ^13^, ^14^, ^15^ Recently, using solid electrolytes to assemble all‐solid‐state lithium‐ion batteries is a promising direction to enable Li metal anode.^2^ For example, the solid electrolyte LiPON, an oxynitride material, was demonstrated to achieve a cycle life of over 10 000 cycles in thin‐film lithium metal batteries.^16^

To protect against the reduction of Li metal, protection coating materials or solid electrolytes that are thermodynamically stable or that form stable passivation layers against Li metal are needed. Many Li binaries such as LiF, Li_2_O, Li_2_S, Li_3_N, and Li_3_P, are thermodynamically stable against Li metal, and some are found in SEI layers.^10^, ^17^, ^18^, ^19^ However, despite that many lithium solid electrolytes were previously reported to be stable against Li metal, multiple experimental and first‐principles studies confirmed the reduction of solid electrolytes, including Li_10_GeP_2_S_12_ (LGPS), NASICON‐type Li_1.3_Al_0.3_Ti_1.7_(PO_4_)_3_ (LATP), and perovskite Li_0.33_La_0.56_TiO_3_ (LLTO).^20^, ^21^, ^22^, ^23^, ^24^, ^25^, ^26^, ^27^ In these materials, Li metal reduces Ge and Ti cations, forming Li—Ge alloy and lithium titanate, respectively. Even for the well‐demonstrated Li‐compatible LiPON materials, recent computational and in situ experimental studies confirmed Li reduction, which leads to a spontaneously formed interphase layer consisting of Li_3_N, Li_2_O, and Li_3_P at Li–LiPON interface.^19^, ^22^ This interphase layer is electronic insulating, passivating LiPON against further Li reduction. Therefore, forming passivating SEI layer against Li metal is critical to achieve good stability. Given the limited number of available materials stable against Li metal, the development of novel Li‐stable protection materials or solid electrolytes is crucial to enable Li metal anode.

However, the design and discovery of Li‐stable materials are still mostly based on a trial‐and‐error approach, due to the lack of knowledge about Li metal stability of different materials chemistry and compositions. Li reduction behavior is only known for a limited number of well‐studied materials. For example, the reduction of Ge and Ti is a known problem in LGPS and LATP, respectively, but it is not clear whether these cations will still be reduced in different compositions or in other anion chemistry. In addition, it is not clear whether the excellent stability of LiPON is a general property of oxynitride chemistry. Such knowledge about Li stability will be crucial in guiding further materials development to stabilize Li metal anode, and will enable guided engineering of materials chemistry to form stable SEI on lithium metal.

In this study, we aim to fill this knowledge gap about Li metal stability and passivation behavior over a wide range of materials chemistry. Using data‐driven first‐principles computation approach based on large‐scale materials database, we studied Li metal stability and lithiation reactions in a wide range of materials chemistry and composition space. We determined the intrinsic thermodynamic stability of materials against Li metal as a function of cation and anion chemistry, and discovered that nitride anion chemistry uniquely exhibits better thermodynamic stability against Li metal compared to oxides, sulfides, and halides. In addition, the materials chemistry and composition range that can form stable passivation interphase against Li metal are identified. On the basis of newly obtained chemistry knowledge from computation, multiple novel strategies to form stable SEI on Li metal anode are proposed, providing opportunities for future research and development of lithium batteries.

## 2 Results

### 2.1 General Trend of Materials Stability against Li

**Figure 1:**
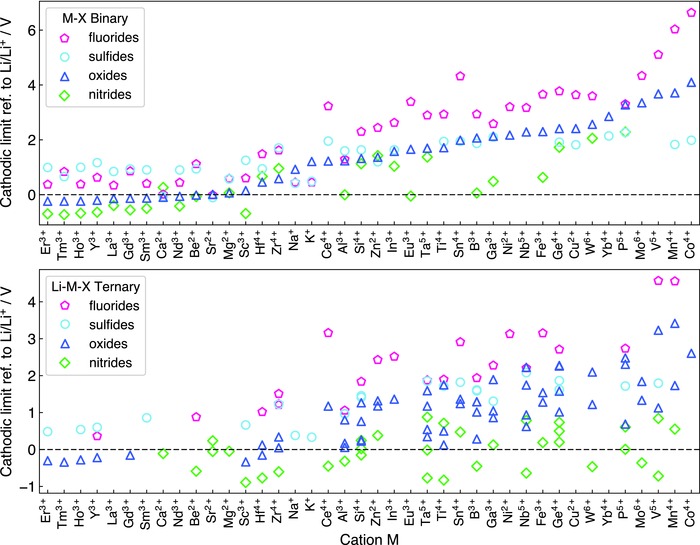
Cathodic limits (referenced to Li/Li^+^) for Li reduction of a) M—X binary compounds and b) Li—M—X ternary compounds in fluoride, sulfide, oxide, and nitride anion chemistries. Only cation M at its highest common valence state is considered. Each data point represents a compound. A full list of compounds and corresponding lithiation reactions are provided in the Supporting Information.

In this study, we considered oxides, sulfides, fluorides, and nitrides, which might be potentially used as solid electrolytes or coating layer materials with Li metal anode. We calculated the Li reduction potential (cathodic limit) of M—X binary compounds and Li—M—X ternary compounds (M = cation) in four anion chemistries, X = N, O, S, and F (**Figure** **1**), which are commonly used in solid electrolytes or coating layer materials. To illustrate our results, we first take Al‐abiding compounds as examples. The binary materials AlF_3_, Al_2_S_3_, and Al_2_O_3_ have high cathodic limits of >1.2 V, and their lithiation reactions with Li metal form Li_9_Al_4_ and Li—X binary materials with a reaction energy of −0.77, −0.74, and −0.23 eV per Li, respectively (**Table** **1**). The same trend is also observed for ternary Li—Al—X (Table S3, Supporting Information), Li—Ge—X, and Li—P—X compounds (Table 1). These examples show that different anion chemistry exhibits different stability against Li metal. In general, fluorides have the highest cathodic limits, yielding poorest stability against Li metal. This is similar to the trend observed in organic liquid electrolytes, as fluorinated compounds usually show higher reduction potential.^7^ Oxides and sulfides have lower cathodic limits than fluorides, and only a few of investigated oxides and sulfides are thermodynamically stable against Li metal.

**Table 1:** Cathodic limits and lithiation reactions for selected fluorides, sulfides, oxides and nitrides. The reaction energy *E*_D_ is for the lithiation of selected compound to from the phase equilibria with Li metal and is normalized to per Li inserted

Compound	Phase equilibria at cathodic limit	Cathodic limit ref. to Li/Li^+^ [V]	Phase equilibria with Li metal	*E*_D_ [eV] per Li
AlN	Li_9_Al_4_, Li_3_AlN_2_	−0.004	Li_9_Al_4_, Li_3_Na)	0.16
Al_2_O_3_	Al, LiAl_5_O_8_	1.23	Li_9_Al_4_, Li_2_O	−0.23
Al_2_S_3_	Al, LiAlS_2_	1.60	Li_9_Al_4_, Li_2_S	−0.74
AlF_3_	Al, Li_3_AlF_6_	1.29	Li_9_Al_4_, LiF	−0.77
Li_5_GeN_3_	Li_15_Ge_4_, Li_3_N	0.20	Li_15_Ge_4_, Li_3_N	−0.20
Li_4_GeO_4_	Ge, Li_2_O	1.02	Li_15_Ge_4_, Li_2_O	−0.72
Li_4_GeS_4_	Ge, Li_2_S	1.62	Li_15_Ge_4_, Li_2_S	−1.04
Li_2_GeF_6_	Ge, LiF	2.71	Li_15_Ge_4_, LiF	−1.60
Li_7_PN_4_	Li_3_P, Li_3_N	0.01	Li_3_P, Li_3_N	−0.01
Li_3_PO_4_	Li_3_P, Li_2_O	0.69	Li_3_P, Li_2_O	−0.69
Li_3_PS_4_	P, Li_2_S	1.72	Li_3_P, Li_2_S	−1.42
LiPF_6_	P, LiF	2.74	Li_3_P, LiF	−2.06

^a)^ Since AlN is stable against Li metal, such phases are not phase equilibria with Li metal, but are fully lithiated products after lithiation with overpotential.

By contrast, nitrides show significant lower cathodic limits compared to other anion chemistry. For example, AlN shows a negative cathodic limit referenced to Li/Li^+^, and a positive reaction energy of 0.16 eV per Li to form Li_9_Al_4_ and Li_3_N after lithiation (Table 1). Therefore, it is thermodynamically unfavorable to lithiate AlN by Li metal. In addition, Li_3_AlN_2_ also shows a negative cathodic limit of −0.32 V (Table S3, Supporting Information) indicating its intrinsic stability against Li metal reduction. These trends are observed in both binary and ternary compounds with other cations. In general, many nitrides have negative reduction potential, and are thermodynamically stable against Li metal. Some of these nitrides that are electronic insulators and decent ionic conductors, e.g., Li_3_AlN_2_, Li_3_BN_2_, Li_5_SiN_3_,^28^ may be used as buffer layer materials to protect against Li metal. Nitride anion chemistry shows unique electrochemical stability at low potentials and against Li metal.

### 2.2 Cation Effect on Li Metal Stability and Passivation

**Figure 2:**
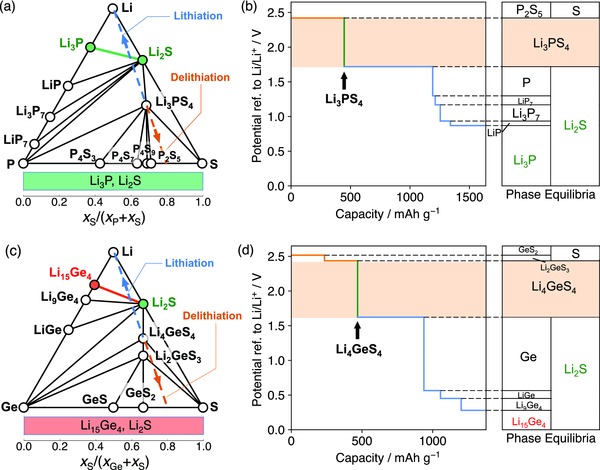
Phase diagrams of a) Li—P—S and c) Li—Ge—S system, and the equilibrium voltage profiles and phase equilibria for lithiation and delithiation reactions of b) Li_3_PS_4_ and d) Li_4_GeS_4_. Li‐stable phases that are electronic insulating (Li_3_P and Li_2_S) and that are electronic conductive (Li_15_Ge_4_) are colored green and red, respectively. The bottom bar in (a) and (c) represents the phase equilibria with Li metal as a function of atomic fraction *x*_S_ and *x*_M_, where passivating and nonpassivating ranges are colored green and red, respectively. The lithiation and delithiation paths in (a) and (c) are marked as blue and orange dashed lines, respectively. These lines represent constant ratio of S and M atomic fraction *x*_S_ to *x*_M_ (M = P, Ge) but varying Li content in the composition.

Using Li—P—S and Li—Ge—S ternary systems as examples, we illustrate the reduction behavior of different cation chemistry and the effect of cation on Li stability and passivation. The Li—P—S composition space (**Figure** **2**a) includes many well‐known solid electrolyte materials, such as Li_3_PS_4_, Li_7_P_3_S_11_, and Li_2_S—P_2_S_5_ glass. For example, the lithiation reaction of Li_3_PS_4_ (blue dashed line in Figure 2a,b) starts with the reduction of P at voltage 1.72 V (Table 1) and eventually leads to Li_3_P and Li_2_S, as

(1) $$\mathrm{8Li}\;+\;{\mathrm{Li}}_{3}{\mathrm{PS}}_{4}\;\to \;{\mathrm{Li}}_{3}P\;+\;{\mathrm{4Li}}_{2}S(\Delta H=\;-\mathrm{11}.\mathrm{39}\;\;\mathrm{eV}\;\mathrm{or}\;-\mathrm{1099}\;\;\mathrm{kJ}\;{\mathrm{mol}}^{-1})$$

which is highly thermodynamically favorable. Since Li_3_P and Li_2_S are the only stable phases against Li among entire Li—P—S composition space, any Li—P—S ternary compound would form the same phase equilibria after full lithiation (green bar in Figure 2a), leading to spontaneous formation of interphase consisting of Li_3_P and Li_2_S. Since both phases are electronic insulating, the formed interphase is passivating against further Li reduction.^22^, ^29^ The formation of Li_3_P and Li_2_S interphase of Li_7_P_3_S_11_ on Li is confirmed by in situ X‐ray photoelectron spectroscopy (XPS) experiments.^30^

Li—Ge—S system (Figure 2c) is commonly used in the design of thio‐LISICON electrolyte. The lithiation reaction of Li_4_GeS_4_ (Figure 2d) would start with the reduction of Ge at 1.62 V (Table 1), continue with Li—Ge alloying reactions, and eventually form Li_15_Ge_4_ and Li_2_S in equilibrium with Li metal (Figure 2d). The entire lithiation reaction can be written as

(2) $$\begin{array}{l} 7.\mathrm{75Li}\;+\;{\mathrm{Li}}_{4}{\mathrm{GeS}}_{4}\;\to \;0.{\mathrm{25Li}}_{\mathrm{15}}{\mathrm{Ge}}_{4}\;+\;{\mathrm{4Li}}_{2}S\\ \;\;\;\;\;\;\;\;\;\;\;\;\;\;\;\;\;\;\;\;\;\;\;\;\;\;\;\;\;\;(\Delta H\;=\;-8.0\;4\;\;\mathrm{eV}\;\mathrm{or}\;-775\;\;\mathrm{kJ}\;{\mathrm{mol}}^{-1}) \end{array}$$

In contrast to Li_3_P, metallic Li_15_Ge_4_ results in the interphase with mixed ionic and electronic conducting (MIEC) property. The simultaneous transport of both Li^+^ and *e* ^−^ through the MIEC interphase allows continuous, favorable Li reduction reaction, and hence cannot passivate at the interface.^22^, ^23^, ^24^, ^25^, ^26^, ^29^, ^31^, ^32^ The nonpassivating behavior of MIEC interphase is confirmed in previous experiments, where the interfacial layers and resistance grow significantly over a short period of time.^26^ Any Li—Ge—S ternary compound leads to the same phase equilibria of Li_15_Ge_4_ and Li_2_S on Li (red bar in Figure 2c). Any Ge‐containing ternary sulfide, regardless of its composition, would not be passivating against Li metal. Therefore, cations play a critical role in forming passivating or nonpassivating interphase layers. Except in nitride anion chemistry, most metal or metalloid cations will be reduced (Figure 1) by Li metal to form metal or Li alloys (Table 1 and Supporting Information), leading to the formation of nonpassivating MIEC interphase. The strategy to protect the reduction of metal or metalloid cations is critical.

### 2.3 Anion Effect on Li Metal Stability and Passivation

**Figure 3:**
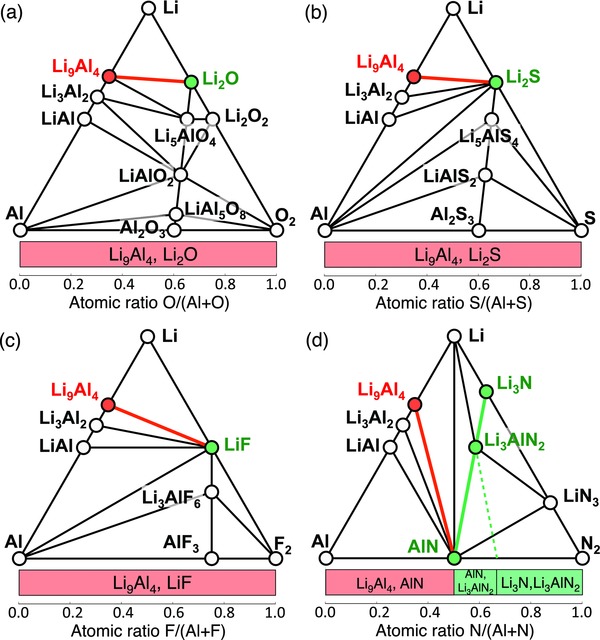
Phase diagrams of a) Li—Al—O, b) Li—Al—S, c) Li—Al—F, d) Li—Al—N systems. Li‐stable phases that are electronic insulating and that are electronic conductive are colored green and red, respectively. The bottom bar represents the phase equilibria with Li metal as a function of anion and cation atomic fraction *x*_X_ and *x*_M_, where passivating and nonpassivating ranges are colored green and red, respectively.

We illustrate the effects of anion chemistry on Li stability and passivation using Li—Al—X (X = O, S, F, and N) ternary systems as examples (Table 1, **Figure** **3**; Table S3, Supporting Information). The lithiation reactions of ternary oxides, sulfides, and fluorides to form Li_9_Al_4_ and Li—X binaries are thermodynamically favorable, leading to spontaneous interphase layer formation (Table S3, Supporting Information). In Li—Al—O system, which is commonly used for coating layer materials,^11^, ^33^, ^34^ the phase equilibria against Li metal are Li_9_Al_4_ alloy and Li_2_O (Table 1 and Figure 3a). Similarly, Li metal phase equilibria of Li—Al—S and Li—Al—F systems also include Li_9_Al_4_ (Table 1 and Figure 3b,c). The presence of metallic Li_9_Al_4_ leads to an MIEC interphase, which may not passivate. These computation results are consistent with the experimental observation of the reduction of Al_2_O_3_ and Al_2_S_3_ at low potentials.^35^, ^36^

The Li—Al—N system shows unique behavior compared to O, S, F‐based systems (Table 1 and Figure 3d). Al‐abiding nitrides AlN and Li_3_AlN_2_ have negative cathodic limit and positive reaction energy for lithiation (Table 1; Table S3, Supporting Information). The phase equilibria with Li metal may consist of different combination of thermodynamically stable phases against Li metal, such as Li_9_Al_4_, AlN, Li_3_AlN_2_, and Li_3_N, depending on the atomic fraction of aluminum *x*_Al_ and nitrogen *x*_N_ in the material composition. When *x*_N_ *< x*_Al_ (red bar in Figure 3d), the phase equilibria of Li reduction are Li_9_Al_4_ and AlN through the reaction

(3) $$\mathrm{Li}\;+\;{\mathrm{Li}}_{x_{\mathrm{Li}}}{\mathrm{Al}}_{x_{\mathrm{Al}}}N_{x_{N}}\;\to \;{\mathrm{Li}}_{9}{\mathrm{Al}}_{4}\;+\;{\mathrm{Li}}_{3}N,$$

leading to nonpassivating MIEC interphase. At higher N content of *x*_Al_ < *x*_N_ < 2*x*_Al_, the formed Li‐stable phase equilibria are AlN and Li_3_AlN_2_, through the reaction

(4) $$\mathrm{Li}\;+\;{\mathrm{Li}}_{x_{\mathrm{Li}}}{\mathrm{Al}}_{x_{\mathrm{Al}}}N_{x_{N}}\;\to \;{\mathrm{Li}}_{3}{\mathrm{AlN}}_{2}\;+\;\mathrm{AlN}.$$

And at even higher N content *x*_N_ > 2*x*_Al_, the phase equilibria are Li_3_AlN_2_ and Li_3_N (Figure 3d), through the reaction

(5) $$\mathrm{Li}\;+\;{\mathrm{Li}}_{x_{\mathrm{Li}}}{\mathrm{Al}}_{x_{\mathrm{Al}}}N_{x_{N}}\;\to \;{\mathrm{Li}}_{3}{\mathrm{AlN}}_{2}\;+\;{\mathrm{Li}}_{3}N.$$

Since Li_3_N, Li_3_AlN_2_, and AlN are electronic insulating,^37^, ^38^, ^39^ the formed interphase would be passivating when *x*_N_ ≥ *x*_Al_ (green bar in Figure 3d). Such passivation interphase between Li metal and Li—Al—N compounds is similar to the spontaneously formed interlayer at the Li–LiPON interface. In addition, both Li_3_AlN_2_ and Li_3_N are ionic conducting materials,^38^, ^39^ which facilitate interfacial ionic transport and reduce interfacial resistance.

Among these four anion chemistry with Al cation, nitride is the only anion chemistry that is stable or can form passivating interphase layer at Li metal contact. This passivation mechanism is activated at high nitrogen content *x*_N_ ≥ *x*_Al_, where electronic insulating nitride phases are formed to passivate the interface. If nitrogen content is low, the formed interphases would still contain Li_9_Al_4_ alloy and cannot passivate (bottom bar in Figure 3d). The passivation is enabled by the electronic insulation of (lithium) metal nitrides, such as Li_3_AlN_2_ and AlN, formed against Li metal. The stability of these nitrides are thermodynamic intrinsic and are unique to nitride. For example, the calculated cathodic limit of ternary nitride Li_3_AlN_2_ is −0.32 V (referenced to Li/Li^+^), which is lower than ternary oxide LiAlO_2_ (0.17 V), and ternary fluoride Li_3_AlF_6_ (1.06 V) (Table S3, Supporting Information).

### 2.4 Stability and Interphase Passivation of Nitrides

**Figure 4:**
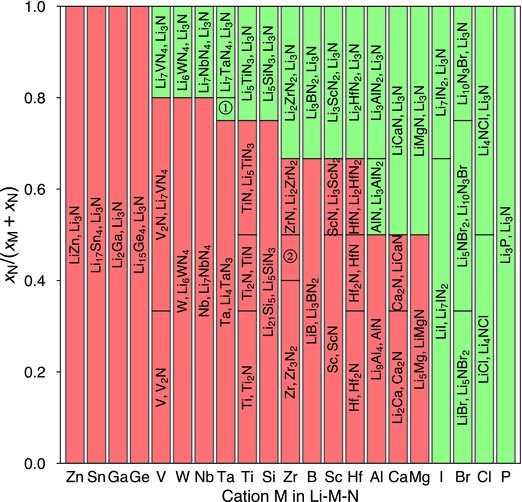
Phase equilibria of Li—M—N compositions in equilibrium with Li metal. The formed products due to Li reduction are a function of atomic fraction *x*_N_ and *x*_M_ (*y* axis) in the composition. (①: [Li_4_TaN_3_, Li_7_TaN_4_]; ②: [Zr_3_N_2_, ZrN].) The passivating and nonpassivating phase equilibria regions are colored green and red, respectively.

We calculated phase equilibria with Li metal for Li—M—N compositions as a function of *x*_M_ and *x*_N_ (**Figure** **4**), according to the reaction

(6) $$\mathrm{Li}\;+\;{\mathrm{Li}}_{x_{\mathrm{Li}}}M_{x_{M}}N_{x_{N}}\;\to \;\mathrm{phase}\;\mathrm{equilibria}\;\mathrm{with}\;\mathrm{Li}\;\mathrm{metal}.$$

The excellent electrochemical stability of metal nitrides against lithium metal is general for many cations (Figure 4). For cation M = Mg, Ca, Al, Hf, Sc, B, Zr, Si, Ti, Ta, Nb, W, and V, passivating interphase may form depending on the material composition. At low N content, the formed interphase includes M metal or Li—M alloy, which are electronic conducting and may not provide passivation. At high N content, the formed interphase consists of Li_3_N and (lithium) metal nitrides (Figure 4). If formed metal nitride is electronic insulating, the interphase would be passivating. These lithium ternary nitrides that are stable against Li metal include LiMgN, LiCaN, Li_3_AlN_2_, Li_2_HfN_2_, Li_3_ScN_2_, Li_3_BN_2_, Li_2_ZrN_2_, Li_5_SiN_3_, Li_5_TiN_3_, Li_4_TaN_3_, Li_7_TaN_4_, Li_7_NbN_4_, Li_6_WN_4_, and Li_7_VN_4_. At high N content, these metal nitrides may form at the interface and may passivate against Li metal.

By contrast, some cations, such as M = Ge, Ga, Sn, and Zn, cannot be stabilized in nitrides against Li metal regardless of the composition. Li‐metal phase equilibria of these Li—M—N systems always contain metal or Li‐metal alloy (Figure 4) forming MIEC interphase. The formed interphase layer is likely not passivating if these cations are used.

The systems with only nonmetal elements, such as M = P, Cl, Br, I, is in general compatible with Li metal. When in equilibrium with Li metal, the reduction products are still electronic insulating phases, including Li_3_P, LiCl, LiBr, and LiI. Some nonmetal elements may change from cation to anion after reduction. In these nitride systems with only nonmetal elements, passivating interphase would form at the Li metal interface regardless of *x*_N_ (Figure 4). Similarly, using only these nonmetal elements also leads to Li‐passivating interphases in oxides and sulfides, as observed in LiPON, Li_3_OCl/Li_3_OBr, Li_9_S_3_N, Li_7_P_3_S_11_, and Li_7_P_2_S_8_I solid electrolytes, which are Li metal compatible.^40^, ^41^, ^42^, ^43^, ^44^

### 2.5 Stability and Interphase Passivation of Mixed‐Anion Nitrides

**Figure 5:**
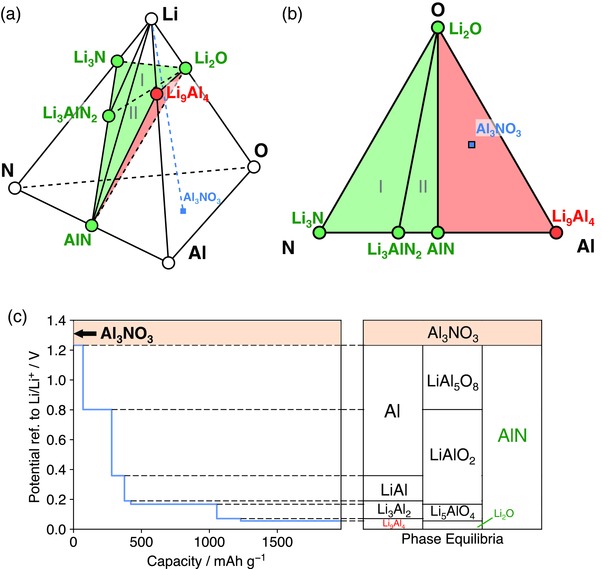
a) Li—Al—O—N quaternary phase diagram showing only Li‐stable phases. The blue dashed line represents the lithiation path of Al_3_NO_3_ (blue square point). b) Grand potential phase diagram of Li—Al—O—N system in equilibrium with Li metal. The Gibbs triangle of the grand potential phase diagram is based on non‐Li composition Al—O—N, which determines the phase equilibria after lithiation. For example, the phase equilibria of Al_3_NO_3_ (blue square) with Li metal are Li_9_Al_4_, Li_2_O, and AlN, corresponding to the red triangle region. The composition regions that form passivating and that form nonpassivating interphases are colored green and red, respectively. c) Voltage profile and phase equilibria for the lithiation of Al_3_NO_3_.

Since nitrides in general show good electrochemical stability against Li metal, here we explore the stability of mixed anion chemistry with nitride corresponding to the doping of nitrogen or nitride into other anion chemistry. We first studied the Li—Al—O—N quaternary system as an example, in which Li—Al—O ternary compound is doped with nitrogen. In the quaternary Li—Al—O—N system, the final phase equilibria in contact with Li metal will only consist of Li‐stable phases, including Li_9_Al_4_, Li_2_O, Li_3_N, AlN, and Li_3_AlN_2_ (**Figure** **5**a). Among those phases, only Li_9_Al_4_ is MIEC, and all other phases are electronic insulating. Similar to the ternary case, the phase equilibria with Li metal are determined by atomic fraction of Al and nitrogen, *x*_N_ and *x*_Al_. At low N content of *x*_N_ *< x*_Al_, the formed phase equilibria consist of Li_9_Al_4_, Li_2_O, AlN represented by the red triangle in Figure 5a,b. For example, the lithiation reaction of Al_3_NO_3_ (gamma‐ALON) follows the blue dashed line in Figure 5a to form these products. This lithiation reaction has the voltage profile and phase equilibria shown in Figure 5c. For compositions at higher N content of *x*_Al_ < *x*_N_ < 2*x*_Al_ (green triangle II in Figure 5), Li phase equilibria are Li_3_AlN_2_, Li_2_O, and AlN. At even higher N content of *x*_N_ > 2*x*_Al_ (green triangle I in Figure 5a,b), the phase equilibria with Li are Li_3_N, Li_2_O, Li_3_AlN_2_. The phase equilibria with Li metal are a function of the Al—O—N composition, and are represented by the grand potential phase diagram in equilibrium with Li metal (Figure 5b). At high N content (green triangle I and II in Figure 5a,b), the formed interphase after lithiation is electronic insulating and hence passivating. These results suggest a strategy of introducing a sufficiently high amount of N to stabilize oxide compounds, which are not stable against Li metal. High‐dose nitrogen doping inhibits the reduction of Al and the formation of Li‐Al alloy, leading to passivating interphase formed against Li metal.

**Figure 6:**
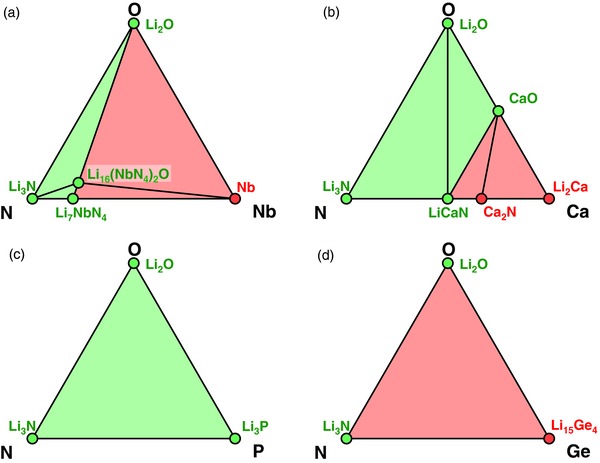
Grand potential phase diagram of a) Li—Nb—O—N, b) Li—Ca—O—N, c) Li—P—O—N, d) Li—Ge—O—N systems in equilibrium with Li metal. The composition regions that form passivating and that form nonpassivating interphases are colored green and red, respectively.

Mixing nitrogen can also stabilize Li—M—O oxides with other cation M = Mg, Ca, Al, B, Zr, Si, Ti, Ta, Nb, V, and W. Similar to M = Al, having high amount of nitrogen in Li—M—O—N systems leads to forming electronic insulating nitrides against Li (**Figure** **6**, **Table** **2**; Supporting Information). For example in Li—Nb—O—N system, electronic insulating phase equilibria form at high N content *x*_N_ ≥ 4*x*_Nb_ (Figure 6a and Table 2). In Li—Ca—O—N system, Li‐stable phase equilibria, including electronic‐insulating LiCaN nitride and CaO oxide, may form as passivation interphase at the high O and N content of *x*_N_ + *x*_O_ ≥ *x*_Ca_ (Figure 6b and Table 2). Therefore, high‐dose nitrogen doping is effective in passivating many oxides with metal cations.

**Table 2:** Composition range in Li—M—N—O systems that form stable passivation interphase

Cation M	Composition range forming passivation interphase layer
Al	*x*_N_ ≥ *x*_Al_
Nb	*x*_N_ ≥ 4*x*_Nb_
B	*x*_N_ ≥ 2*x*_B_
Zr	*x*_N_ ≥ 2*x*_Zr_
Ta	*x*_N_ ≥ 4*x*_Ta_ or *x*_N_ ≥ 3*x*_Ta_ + 2*x*_O_
Ti	*x*_N_ ≥ 3*x*_Ti_
Si	*x*_N_ ≥ 3*x*_Si_
V	*x*_N_ ≥ 4*x*_V_
W	*x*_N_ ≥ 4*x*_W_
Mg	*x*_N_ ≥ *x*_Mg_
Ca	*x*_N_ + *x*_O_ ≥ *x*_Ca_
P, Cl, Br, I	Any *x*_N_ and *x*_M_
Ge, Sn, Ga, Zn	None

Li—P—O—N oxynitride system has Li phase equilibria consisting of only electronic insulating phases, Li_3_N, Li_2_O, and Li_3_P (Figure 6c), which are observed by in situ XPS experiments.^19^ In addition, Li_3_N and Li_3_P are fast lithium ionic conductors, which facilitate interfacial Li transport and reduce interface resistance. The formation of passivating interphase results in excellent Li compatibility of LiPON. Similarly, other nonmetal elements, such as Cl, Br, and I, also always form passivating interphase at Li metal. Such passivating layer, as in the case of Li‐LiPON interface, may achieve good stability on Li metal anode.

Unfortunately, for oxides with some other cations, passivating interphases cannot be achieved by the introduction of nitrogen. For example, the phase equilibria of Li—Ge—O—N system with Li metal are always Li_3_N, Li_2_O, and Li_15_Ge_4_ regardless of N content (Figure 6d). The presence of Li_15_Ge_4_ would lead an MIEC interphase. Similarly, materials containing Sn, Ga, or Zn have Li phase equilibria always containing metal or Li‐metal alloy (Figure S1, Supporting Information and Table 2), and hence may not be passivated by the formation of nitrides.

## 3 Discussion

Our computation results revealed the Li metal stability of materials with different cation and anion chemistry. Most oxides, sulfides, and fluorides, which are widely studied as current solid electrolytes or coating layer materials, are not stable against Li metal. Metal and metalloid cations in oxides, sulfides, and fluorides (and other halides) are reduced at low potentials in most of these compounds. The thermodynamically favorable Li reduction of metal cations would lead to the formation of MIEC interphase and continuous decomposition of bulk materials, resulting in poor stability against Li metal. To avoid the detrimental effects of metal cations on stability, using only nonmetal elements, such as P, Cl, Br, I, instead forms passivating interphase and enables stability against Li metal, as demonstrated in some Li‐compatible solid electrolytes, such as LiPON, Li_3_OCl/Li_3_OBr, Li_9_S_3_N, Li_7_P_3_S_11_, and Li_7_P_2_S_8_I.^40^, ^41^, ^42^, ^43^, ^44^ However, there is a limited choice of these compounds for stabilizing Li metal. Our results provide the composition space to select, design, and discover materials that are stable against Li.

The major discovery of our study is that nitride anion chemistry has significantly better stability against Li metal compared to oxide, sulfide, and halide. Many nitride materials are thermodynamically stable against Li metal. In addition, the nitrides formed at the interface during lithiation become stable, passivating SEI against Li metal. This chemistry knowledge from our computation suggests multiple strategies as follows to stabilize materials against Li metal and to improve the performance of Li metal anode.

(1) Many nitride materials that are electronic insulators may be used as protective buffer layer materials on Li metal anode. These buffer layers are thermodynamically stable against Li metal, thus will protect the electrolytes from reduction and will have long‐term stability on Li metal. For example, Li_3_N is demonstrated as an effective buffer layer material to protect liquid or solid electrolyte and to improve the cyclability and coulombic efficiency of Li metal anode.^11^, ^12^, ^13^ Besides Li_3_N, many Li‐containing nitrides that are stable against Li metal, e.g., Li_3_AlN_2_, Li_5_SiN_3_, Li_3_BN_2_, LiMgN, LiCaN, Li_2_HfN_2_, Li_3_ScN_2_, Li_2_ZrN_2_, Li_5_TiN_3_, Li_4_TaN_3_, Li_7_TaN_4_, Li_7_NbN_4_, Li_6_WN_4_, and Li_7_VN_4_, may also be used as buffer layers on Li metal anode. In addition, developing new materials, such as Li_18_P_6_N_16_ nitridophosphate,^45^ may also be a promising direction.
(2) Our results also suggest that high‐dose nitrogen doping in materials can lead to in situ spontaneous passivation against Li metal. Surface modification techniques such as nitrogen ion/plasma treatment and other nitriding techniques can locally enrich nitrogen content on the surfaces of the material. The nitrogen‐rich surface put into contact with Li metal would form nitride passivation interphase layers and woudl be transformed into a Li‐stable interface. For example, if one can introduce sufficient amount of N into the surface region of Al_2_O_3_ to the composition range *x*_N_ ≥ *x*_Al_, a passivation interphase of Li_3_AlN_2_/AlN and Li_2_O would form (Figure 5). The necessary nitrogen content and composition to passivate each cation is provided in our calculations (Figure 6, Table 2; Figure S1, Supporting Information). Various experimental approaches can be used to introduce nitrogen into the material and to stabilize the material interfaces against Li metal. For example, applying nitrogen‐doped thin films on Li metal anodes has shown to improve Li metal stability and to inhibit lithium dendrite.^46^ Multiple studies also demonstrated nitrogen doping on carbon‐based anode materials greatly improves the cycling stability.^47^, ^48^, ^49^
(3) Another way to locally enrich nitrogen is introducing Li_3_N to further react at the interface and to form a stable SEI layer. Li_3_N may be introduced as a coating layer or by nitriding Li metal. For example, Li_3_N can react with Al_2_O_3_ at the contact interface and form stable SEI consisting Li_3_AlN_2_ and Li_2_O through an exothermic reaction, Al_2_O_3_ + 4 Li_3_N → 2 Li_3_AlN_2_ + 3 Li_2_O (Δ*H* = −5.76 eV or −556 kJ mol^−1^). A similar strategy was demonstrated on Li metal protection by Visco et al.^11^
(4) In addition, mixing nitrogen‐containing compounds, such as nitride, nitrite, and nitrate, into the materials can also stabilize the interface. For example, Al_2_O_3_ mixed with LiNO_3_ may also form an SEI of Li_3_AlN_2_ and Li_2_O in contact with Li through an exothermic reaction Al_2_O_3_ + 4 LiNO_3_ + 32 Li → 2 Li_3_AlN_2_ + 15 Li_2_O (Δ*H* = −58.20 eV or −5615 kJ mol^−1^). A similar strategy of using nitrogen‐containing additives in liquid electrolytes was demonstrated in forming stable SEI layer on Li metal anode.^50^, ^51^, ^52^, ^53^, ^54^ For example, LiNO_3_ as additive was reported to significantly improve the cyclability and coulombic efficiency of Li metal anode by forming an SEI containing lithium nitride and oxynitrides.^55^, ^56^, ^57^ In agreement with our prediction, high concentration of LiNO_3_ is needed to achieve high nitrogen content and to form a stable SEI.^52^ In addition, other nitrogen‐containing compounds, such as cyanide and cyanate, are demonstrated for nitriding metals. Novel nitrogen‐containing compounds may be developed for nitriding materials in lithium batteries.

While our computation demonstrated the thermodynamic origin of using nitrides and rich nitrogen content to stabilize Li metal anode, experimental techniques to realize these new strategies in lithium batteries may need further study and to address multiple potential challenges. First, nitride buffer coating layer is desired to be thin (≈10 nm), dense, and uniform, to achieve low interfacial resistance and good passivation.^58^ Applying such coating may requires thin‐film‐based techniques, such as sputtering, physical/chemical vapor deposition, atomic layer deposition, and plasma nitriding, which may not be cost competitive.^59^ In addition, some nitrides may be moisture or air sensitive,^58^ which requires additional protection during manufacturing and processing. Gas nitriding techniques based on nitrogen rich gas, such as ammonia, may be an economic and scalable approach as demonstrated in the nitriding of metals.^59^ However, ammonia treatment may induce H into the materials, which may be detrimental for the properties of electrolytes or electrodes. Experimental techniques to economically coat nitride layers and to effectively dope high nitrogen content at the interface need further research and development.

The aforementioned strategies of using nitrogen anion chemistry to stabilize Li metal anode may offer multiple additional advantages. The thermodynamically favorable lithiation reactions provide interfacial wetting, which would promote interfacial contacts.^23^, ^29^, ^60^ Moreover, a fraction of nitrogen may be lithiated to form Li_3_N, a good ionic conductor, as part of the interphase to facilitate interfacial Li ion transport. In addition, many nitrides exhibit good tolerance to stress, which is a desirable mechanical property for stable interfacial layer during cycling and for suppressing Li dendrite growth.^59^, ^61^, ^62^, ^63^, ^64^, ^65^ Therefore, the formation of stable SEI layer using nitride anion chemistry provides good stability against Li metal, reduces interfacial resistance, and may mechanically suppress Li dendrite formation. High nitrogen content as predicted by our calculations (Figure 6 and Table 2) is necessary to form phase equilibria of only passivating phases and to completely inhibit the formation of reduced metallic phases, while a lower nitrogen content may still retard interfacial degradation and benefit the performance of Li metal anode as a result of kinetic stabilization.

The limitations of using the thermodynamic scheme to predict interfacial phenomena should be noted as follows. In this study, we approximated the interphase as the phase equilibria, which are the most thermodynamically favorable states, whereas kinetics of reaction and diffusion are not considered. While kinetics and diffusion play crucial roles in the reactions at the interfaces, the thermodynamics provide an ultimate boundary of stability. Given facile Li diffusion, it is expected the phase equilibria would form at the immediate contact with Li metal, as demonstrated in previous in situ XPS study.^19^, ^24^, ^25^, ^26^, ^30^ Despite possible kinetic or diffusional limitations for such reactions, the thermodynamic driving force would always exist for forming the most favorable phase equilibria. These decomposition reactions may have a significant impact on the long‐term stability of the battery. It is possible that the formed interphase layers are different from thermodynamic equilibria. For example, some phases that are not stable against lithium metal may form due to kinetic stabilization. In addition, kinetic limitation of the lithiation reduction at the interface may lead to the formation of intermediate decomposition products, which may be passivating to slow down further degradation.^30^ As a result of such kinetic effects, some intermediate decomposition products or less than adequate nitrogen doping may still improve the stability at Li metal interfaces. Nevertheless, designing the interface with thermodynamic intrinsic stability against Li metal is still desired to realize long‐term stability, good cyclability, and high coulombic efficiency of the lithium battery.

Additional approximations made in our computation scheme and their potential impact are noted as follows. We used the mixture of bulk phase properties to approximate the interphase property, and did not consider the microstructures of interphase. For example, if the formed electronic‐conducting phases are not percolating from Li metal to electrolyte, the overall interphase layer would still be passivating. The effects of microstructures, local off‐stoichiometry, defects accumulation, and kinetically stabilized metastable phases, which were not considered in our calculations, may also impact interphase properties and passivation effects. In addition, our scheme relies on known phases to predict Li reduction products of materials. There may be errors in the predicted phase equilibria if some phases that exist in nature were not included. This scenario is more likely in nitride systems, which have been relatively less studied than oxides. In addition, due to the lack of available electronic conductivity data of many materials, we used a simple cutoff based on density functional theory (DFT) calculated band gap and the valence states of metal cations to judge whether a phase is electronic conductive or insulating. While the trend of the nitride stability is captured, using experimentally measured electronic conductivity would be more reliable in estimating the interphase properties. Some passivating interphase may still have a low electronic conductivity leading to a slow interphase layer growth and an impedance increase during cycling or over time. For example, while the SEI layer on graphite anode in commercial lithium ion battery is well accepted as a passivation film, Dahn and co‐workers showed slow growth of SEI and continuous lithium loss.^66^, ^67^ The Li‐compatible solid electrolyte Li_7_P_3_S_11_, also shows a slow interphase impedance increase over time.^30^ Nevertheless, these passivating interphases have significantly better stability than nonpassivating interphases, and provide improved cyclability and battery performance.^26^

## 4 Conclusion

In this study, our computation revealed that material stability against lithium metal is governed by cation and anion chemistry. Metal cations in oxides, sulfides, and fluorides are usually reduced by lithium metal, leading to poor stability against lithium metal. We discovered that nitride anion chemistry shows unique stability against Li metal. In general, metal nitrides have a significantly lower reduction potential than oxides, sulfides, and fluorides, and many nitrides are thermodynamically stable against Li metal. On the basis of this chemistry knowledge, we suggest new strategies to form stable SEI on Li metal anode. Many nitrides that have intrinsic Li metal stability can be used as buffer layers to protect materials against Li metal. In addition, high‐dose nitrogen doping and nitrogen enriching at the interface can lead to spontaneous formation of a stable, passivating SEI on Li metal, transforming a lithium unstable interface into a lithium‐stable interface. Specifically, many cations, such as Mg, Ca, B, Al, Zr, V, W, Si, Ti, Nb, and Ta, can be protected in nitride anion chemistry systems at sufficiently high nitrogen content. However, some cations, such as Ge, Sn, Ga, and Zn, are always reduced by Li metal regardless of anion chemistry and composition. Our results provide guiding principles for the selection, design, and discovery of materials with Li metal stability, and predict interfacial engineering strategies to stabilize Li metal anodes in lithium batteries.

## 5 Experimental Section

Most compounds were from the Materials Project (MP)^68^ and Inorganic Crystal Structure Database, and 21 additional lithium ternary compounds were predicted using substitution prediction algorithm developed by Hautier et al.^69^ (Details are provided in the Supporting Information.) The energies of most materials were obtained from the MP database. For materials that are not available in the MP database, DFT computation based on Perdew–Burke–Ernzerhof generalized gradient approximation (GGA) functionals^70^ described by the projector augmented‐wave approach^71^ as implemented in the Vienna Ab initio Simulation Package^72^ were performed. The parameters of DFT calculations, such as the plane‐wave energy cutoff and *k*‐points density, were consistent with those used for the MP.^73^ The energy correction schemes for transition metal, O, S, N, and F elements were applied as in the MP.^74^, ^75^ The calculated reaction energies and voltages were based on DFT energies as in previous studies,^20^, ^76^ where the contributions of *PV* terms and entropy terms were neglected.

The electrochemical stability of materials was investigated using the same method as in the previous studies.^20^, ^22^, ^29^ The phase diagram was constructed by the convex hull of the energies of all phases in given composition space (e.g., Li—M—X) using *pymatgen*.^77^ As a result, only the lowest energy (thermodynamically most favorable) phases for any given composition are presented in the phase diagram. As in our previous study, the applied electrostatic potential φ was considered in Li chemical potential as^22^, ^29^

(7) $$\mu_{\mathrm{Li}}\;\left(\phi \right)\;=\;\mu_{\mathrm{Li}}^{0}\;-\;e\phi$$

Thermodynamic phase equilibria, i.e., the phases with the lowest energy at given composition, as a function of different potentials were obtained from grand potential phase diagrams at varying Li chemical potential.^77^

Due to the lack of electronic conductivity data from other sources, the valence state of transition metal cations and the band gap calculated in GGA were used to judge whether a phase is electronic insulating. Phases that have transition metal cations at their highest valence state and that have a calculated band gap wider than 0.7 eV in GGA are considered electronic insulating. Though the band gap calculated in GGA was systematically underestimated, cut‐off value was chosen as 0.7 eV, which was the GGA calculated gap of Li_3_P, a known poor electronic conductor.^26^, ^78^

## Supporting information

Supplementary
