# Supplementary material for: Strategies Based on Nitride Materials Chemistry to Stabilize Li Metal Anode
Source: Adv Sci (Weinh). 2017 Mar 3;4(8):1600517. doi: 10.1002/advs.201600517 (PMC5566245; doi:10.1002/advs.201600517)
Supplement: Supplementary file 1 — Supplementary [file ADVS-4-na-s001.pdf]

Copyright WILEY-VCH Verlag GmbH & Co. KGaA, 69469 Weinheim, Germany, 2017.

Supporting Information

for *Adv. Sci.,* DOI: 10.1002/advs.201600517

Strategies Based on Nitride Materials Chemistry to Stabilize Li Metal Anode

*Yizhou Zhu, Xingfeng He, and Yifei Mo**

Supporting Information

Strategies Based on Nitride Materials Chemistry to Stabilize Li Metal Anode

Yizhou Zhu [1], Xingfeng He [1], Yifei Mo [1,2] *

[1] Department of Materials Science and Engineering,

[2] University of Maryland Energy Research Center,

University of Maryland, College Park, MD 20742

* Email: yfmo@umd.edu

1. List of Compounds.

The lithium ternary compounds Li-M-X (X = N, O, S, F) investigated in Fig. 1 are summarized in Table S1. The structures and energies of most compounds were obtained from the Materials Project (MP) database. We added 26 additional lithium ternary compounds (Table S2), of which 21 were predicted using substitution prediction algorithm and 5 were from Inorganic Crystal Structure Database (ICSD).

Table S1. Lithium ternary compounds Li-M-X (X = N, O, S, F)

Cation	Ternary Compounds Li-M-X	Cation	Ternary Compounds Li-M-X
Er^{3+}	$LiErO_2$, $LiErS_2$	Tm^{3+}	$LiTmO_2$
Ho^{3+}	$LiHoO_2$, $LiHoS_2$	Y^{3+}	$LiYO_2$, $LiYS_2$, $LiYF_4$
Gd^{3+}	$LiGdO_2$	Sm^{3+}	$LiSmS_2$
Ca^{2+}	LiCaN	Be^{2+}	LiBeN, Li_2BeF_4
Sr^{2+}	LiSrN, Li_4SrN_2	Mg^{2+}	LiMgN
Sc^{3+}	Li_3ScN_2, $LiScO_2$, $LiScS_2$	Hf^{4+}	Li_2HfN_2, Li_2HfO_3, $Li_6Hf_2O_7$, Li_2HfF_6
Zr^{4+}	Li_2ZrN_2, $Li_6Zr_2O_7$, Li2ZrO$_3$, Li_2ZrS_3, Li_4ZrF_8, Li_2ZrF_6, $Li_3Zr_4F_{19}$	Na^{+}	LiNaS
K^{+}	LiKS	Ce^{4+}	Li_2CeN_2, Li_2CeO_3, $LiCeF_5$
Al^{3+}	Li_3AlN_2, $LiAl_5O_8$, $LiAlO_2$, Li_5AlO_4, $LiAlS_2$, Li_5AlS_4, Li_3AlF_6	Si^{4+}	Li_5SiN_3, Li_2SiN_2, $LiSi_2N_3$ Li_2SiO_3, $Li_2Si_2O_5$, Li_8SiO_6, Li_4SiO_4, Li_8SiS_6, Li_2SiS_3, Li_2SiF_6
Zn^{2+}	LiZnN, $Li_{10}Zn_4O_9$, Li_6ZnO_4, Li_2ZnF_4	In^{3+}	$LiInO_2$, $LiInF_4$
Ta^{5+}	Li_4TaN_3, Li_7TaN_4, $LiTaN_2$, $LiTa_3O_8$, Li_5TaO_5, $LiTaO_3$, Li_3TaO_4, Li_3TaS_4, $LiTaF_6$	Ti^{4+}	Li_2TiN_2, Li_5TiN_3, Li_4TiO_4, Li_2TiO_3, $Li_4Ti_5O_{12}$, Li_2TiS_3, Li_2TiF_6
Sn^{4+}	Li_2SnN_2, Li_8SnO_6, Li_2SnO_3, Li_2SnS_3, Li_2SnF_6	B^{3+}	Li_3BN_2, $Li_2B_4O_7$, $LiBO_2$, Li_3BO_3, $Li_3B_7O_{12}$, Li_3BS_3, $Li_5B_7S_{13}$, $LiBF_4$
Ga^{3+}	Li_3GaN_2 $LiGaO_2$, Li_5GaO_4, $LiGa_5O_8$, $LiGaS_2$, Li_3GaF_6	Ni^{2+}	Li_2NiF_4
Nb^{5+}	Li_7NbN_4, $LiNbN_2$, $LiNbO_3$, $LiNb_3O_8$, Li_3NbO_4, $Li_8Nb_2O_9$ Li_3NbS_4, $LiNbF_6$	Fe^{3+}	Li_3FeN_2, $LiFeO_2$, Li_5FeO_4, Li_3FeF_6
Ge^{4+}	Li_5GeN_3, Li_2GeN_2, $LiGe_2N_3$, $Li_2Ge_7O_{15}$, Li_4GeO_4, $Li_4Ge_5O_{12}$, Li_2GeO_3 Li_2GeS_3, Li_4GeS_4, Li_2GeF_6	W^{6+}	Li_6WN_4, Li_2WO_4, Li_4WO_5
P^{5+}	$LiPN_2$, Li_7PN_4, $Li_4P_2O_7$, $LiPO_3$, Li_3PO_4, Li_3PS_4, $LiPF_6$	Mo^{6+}	Li_6MoN_4, Li_2MoO_4, Li_4MoO_5
V^{5+}	Li_7VN_4, $LiVN_2$, Li_3VO_4, $LiVO_3$, Li_3VS_4, $LiVF_6$	Mn^{4+}	$LiMn_2N_3$, Li_2MnN_2, Li_2MnO_3, $Li_4Mn_5O_{12}$, Li_2MnF_6
Co^{4+}	Li_8CoO_6		

Table S2. Additional lithium ternary compounds.

Compound	Form. E (eV/atom)	Spacegroup	Structure source
$LiAlS_2$	-1.614	$P4_12_12$	S-O substitution from $LiAlO_2$ (ICSD-23815)
Li_5AlS_4	-1.583	Pcab	S-O substitution from Li_5AlO_4 (ICSD-42697)
Li_8SiS_6	-1.496	$P6_3cm$	S-O substitution from Li_8SiO_6 (ICSD-65176)
Li_2SiS_3	-1.385	$Ccm2_1$	S-O substitution from Li_2SiO_3 (ICSD-853)
Li_2SiF_6	-3.423	P321	ICSD-425923
Li_2GeN_2	-0.609	P-3m1	Ge-Zr substitution from Li_2ZrN_2 (ICSD-78790)
$LiGe_2N_3$	-0.516	$Cmc2_1$	Ge-Si substitution from $LiSi_2N_3$ (ICSD-34118)
Li_2GeS_3	-1.218	$Ccm2_1$	S-O substitution from Li_2GeO_3 (ICSD-100403)
Li_2SnN_2	-0.403	P-3m1	Sn-Zr substitution from Li_2ZrN_2 (ICSD-78790)
Li_2SnS_3	-1.211	C2/c	S-O substitution from Li_2SnO_3 (ICSD-21032)
Li_2SnF_6	-3.043	P-31m	Sn-Zr substitution from Li_2ZrF_6 (ICSD-2644)
Li_2TiN_2	-1.194	P-3m1	Ti-Zr substitution from Li_2ZrN_2 (ICSD-78790)
Li_2TiS_3	-1.668	C2/c	S-O substitution from Li_2TiO_3 (ICSD-15150)
Li_2ZrS_3	-1.800	C2/c	S-O substitution from Li_2ZrO_3 (ICSD-94893)
$LiNbN_2$	-1.035	R-3m	Li-Na, Nb-Ta substitution from $NaTaN_2$ (ICSD-67347)
$LiNbF_6$	-3.414	R-3	ICSD-165202
$LiTaN_2$	-1.233	R-3m	Li-Na substitution from $NaTaN_2$ (ICSD-67347)
Li_3TaS_4	-1.526	C2/c	S-O substitution from Li_3TaO_4 (ICSD-281301)
$LiTaF_6$	-3.598	R-3	ICSD-165205
Li_2ZnF_4	-2.941	Fd-3m	Zn-Ni substitution from Li_2NiF_4 (ICSD-202859)
Li_2HfF_6	-3.788	P-31m	ICSD-251074
Li_2HfN_2	-1.353	P-3m1	Hf-Zr substitution from Li_2ZrN_2 (ICSD-78790)
LiCeF5	-3.830	$I4_1/a$	ICSD-426211
$LiVN_2$	-1.024	$Pna2_1$	Li-Mg, V-Si substitution from $MgSiN_2$ (ICSD-23501)
$LiMn_2N_3$	-0.496	$Cmc2_1$	Mn-Si substitution from $LiSi_2N_3$ (ICSD-78790)
Li_2MnN_2	-0.663	Pbca	Mn-Si substitution from Li_2SiN_2 (ICSD-420126)

2. Lithiation/delithiation reaction of materials

Table S3. Lithiation and delithiation reactions of typical Al-abiding ternary nitride, oxide, sulfide and fluoride.

a) Li_3AlN_2

Potential ϕ (V) ref. to Li/Li$^+$	μ_{Li} (eV) ref. to Li metal	Δn_{Li} per formula	Phase equilibria
-0.316	0.316	5.25	Li_9Al_4, Li_3N
/	/	0	Li_3AlN_2
0.789	-0.789	-2.667	AlN, LiN_3
1.668	-1.668	-3	AlN, N_2

b) $LiAlO_2$

Potential ϕ (V) ref. to Li/Li$^+$	μ_{Li} (eV) ref. to Li metal	Δn_{Li} per formula	Phase equilibria
0.056	-0.056	5.25	Li_9Al_4, Li_2O
0.071	-0.071	2.625	Li_9Al_4, Li_5AlO_4
0.167	-0.167	2.25	Li_3Al_2, Li_5AlO_4
/	/	0	$LiAlO_2$
3.697	-3.697	-0.8	$LiAl_5O_8$, O_2
4.101	-4.101	-1	Al_2O_3, O_2

c) $LiAlS_2$

Potential ϕ (V) ref. to Li/Li$^+$	μ_{Li} (eV) ref. to Li metal	Δn_{Li} per formula	Phase equilibria
0.071	-0.071	5.25	Li_9Al_4, Li_2S
0.190	-0.190	4.5	Li_3Al_2, Li_2S
0.359	-0.359	4	LiAl, Li_2S
0.963	-0.963	3	Al, Li_2S
0.973	-0.973	1.5	Li_5AlS_4, Al
/	/	0	$LiAlS_2$
2.812	-2.812	-1	Al_2S_3, S

d) Li_3AlF_6

Potential ϕ (V) ref. to Li/Li$^+$	μ_{Li} (eV) ref. to Li metal	Δn_{Li} per formula	Phase equilibria
0.071	-0.071	5.25	Li_9Al_4, LiF
0.190	-0.190	4.5	Li_3Al_2, LiF
0.359	-0.359	4	LiAl, LiF
1.057	-1.057	3	LiF, Al
/	/	0	Li_3AlF_6
6.477	-6.477	-3	AlF_3, F_2

Table S4. Cathodic limits and lithiation reactions for all binary and ternary fluorides, sulfides, oxides and nitrides plotted in Fig. 1. The reaction energy E_D normalized to per Li inserted is for the lithiation of selected compound to from the phase equilibria with Li metal. For materials that are stable against Li metal (i.e., with negative cathodic limit), the "Phase equilibria at Li" column lists the fully lithiated products after lithiation with overpotential.

**Compound**	**Cathodic limit /V ref. to Li/Li$^+$**	**Phase equilibria at cathodic limit**	**Phase equilibria at Li**	**E_D /eV norm. to Li**
ErN	-0.699	Li_3N, Er	Li_3N, Er	0.699
ErF_3	0.371	Er, LiF	LiF, Er	-0.371
Er_2S_3	0.991	$LiErS_2$, ErS	Er, Li_2S	-0.284
$LiErS_2$	0.484	Li_2S, ErS	Er, Li_2S	-0.200
Er_2O_3	-0.239	$LiErO_2$, Er	Er, Li_2O	0.288
$LiErO_2$	-0.304	Er, Li_2O	Er, Li_2O	0.304
TmN	-0.728	Li_3N, Tm	Li_3N, Tm	0.728
TmF_3	0.837	Tm, LiF	LiF, Tm	-0.837
Tm_2S_3	0.660	TmS, Li_2S	Li_2S, Tm	-0.276
Tm_2O_3	-0.238	$LiTmO_2$, Tm	Tm, Li_2O	0.316
$LiTmO_2$	-0.342	Li_2O, Tm	Tm, Li_2O	0.342
HoN	-0.675	Li_3N, Ho	Li_3N, Ho	0.675
HoF_3	0.378	LiF, Ho	LiF, Ho	-0.378
Ho_2S_3	0.997	$LiHoS_2$, HoS	Ho, Li_2S	-0.280
$LiHoS_2$	0.541	Li_2S, HoS	Ho, Li_2S	-0.204
Ho_2O_3	-0.237	$LiHoO_2$, Ho	Ho, Li_2O	0.270
$LiHoO_2$	-0.280	Ho, Li_2O	Ho, Li_2O	0.280
YN	-0.639	Y, Li_3N	Li_3N, Y	0.639
YF_3	0.625	$LiYF_4$, Y	Y, LiF	-0.429
$LiYF_4$	0.364	Y, LiF	LiF, Y	-0.364
Y_2S_3	1.167	Y_5S_7, $LiYS_2$	Y, Li_2S	-0.278
$LiYS_2$	0.600	Li_2S, YS	Y, Li_2S	-0.209
Y_2O_3	-0.204	$LiYO_2$, Y	Y, Li_2O	0.215
$LiYO_2$	-0.219	Y, Li_2O	Y, Li_2O	0.219
LaN	-0.398	Li_3N, La	Li_3N, La	0.398
LaF_3	0.339	La, LiF	LiF, La	-0.339
La_2S_3	0.846	LaS, Li_2S	La, Li_2S	-0.174
La_2O_3	-0.139	La, Li_2O	La, Li_2O	0.139
GdN	-0.559	Li_3N, Gd	Li_3N, Gd	0.559
GdF_3	0.856	LiF, Gd	LiF, Gd	-0.856
Gd_2S_3	0.935	GdS, Li_2S	Li_2S, Gd	-0.281
Gd_2O_3	-0.132	$LiGdO_2$, Gd	Li_2O, Gd	0.149

$LiGdO_2$	-0.154	Gd, Li_2O	Li_2O, Gd	0.154
SmN	-0.500	Li_3N, Sm	Li_3N, Sm	0.500
SmF_3	0.404	LiF, Sm	Sm, LiF	-0.404
Sm_2S_3	0.906	$LiSmS_2$, SmS	Sm, Li_2S	-0.283
$LiSmS_2$	0.858	Li_2S, SmS	Sm, Li_2S	-0.275
Sm_2O_3	-0.126	Sm, Li_2O	Li_2O, Sm	0.126
Ca_3N_2	0.263	Ca_2N, LiCaN	Li_3N, Li_2Ca	0.075
LiCaN	-0.111	Li_3N, Li_2Ca	Li_2Ca, Li_3N	0.111
CaF_2	0.009	LiF, Li_2Ca	Li_2Ca, LiF	-0.009
CaS	-0.061	Li_2Ca, Li_2S	Li_2S, Li_2Ca	0.061
CaO	-0.096	Li_2Ca, Li_2O	Li_2Ca, Li_2O	0.096
NdN	-0.418	Li_3N, Nd	Li_3N, Nd	0.418
NdF_3	0.441	Nd, LiF	LiF, Nd	-0.441
Nd_2S_3	0.900	Li_2S, NdS	Li_2S, Nd	-0.288
Nd_2O_3	-0.059	Nd, Li_2O	Nd, Li_2O	0.059
Be_3N_2	-0.069	LiBeN, Be	Be, Li_3N	0.414
LiBeN	-0.587	Be, Li_3N	Li_3N, Be	0.587
BeF_2	1.119	Li_2BeF_4, Be	Be, LiF	-0.999
Li_2BeF_4	0.879	Be, LiF	Be, LiF	-0.879
BeS	0.943	Li_2S, Be	Be, Li_2S	-0.943
BeO	-0.015	Be, Li_2O	Be, Li_2O	0.015
SrLiN	0.237	$SrLi_4N_2$, Sr_2N	Li_3N, Sr_3Li_2	0.011
$SrLi_4N_2$	-0.054	Sr_3Li_2, Li_3N	Li_3N, Sr_3Li_2	0.054
SrF_2	-0.013	Sr_3Li_2, LiF	Sr_3Li_2, LiF	0.013
SrS	-0.105	Sr_3Li_2, Li_2S	Sr_3Li_2, Li_2S	0.105
SrO	0.013	Li_2O, Sr	Li_2O, Sr_3Li_2	-0.011
Mg_3N_2	0.064	$LiMg_2$, LiMgN	Li_3N, Li_5Mg	0.022
LiMgN	-0.047	Li_3N, Li_5Mg	Li_3N, Li_5Mg	0.047
MgF_2	0.585	Mg, LiF	Li_5Mg, LiF	-0.186
MgS	0.575	Mg, Li_2S	Li_5Mg, Li_2S	-0.184
MgO	0.065	$LiMg_2$, Li_2O	Li_2O, Li_5Mg	-0.030
ScN	-0.689	Li_3ScN_2, Sc	Sc, Li_3N	0.790
Li_3ScN_2	-0.892	Li_3N, Sc	Li_3N, Sc	0.892
ScF_3	0.600	Sc, LiF	Sc, LiF	-0.600
Sc_2S_3	1.252	$LiScS_2$, ScS	Sc, Li_2S	-0.410
$LiScS_2$	0.664	ScS, Li_2S	Sc, Li_2S	-0.312
Sc_2O_3	0.148	$LiScO_2$, Sc	Sc, Li_2O	0.215
$LiScO_2$	-0.336	Sc, Li_2O	Li_2O, Sc	0.336
Hf_3N_4	0.667	HfN, Li_2HfN_2	Li_3N, Hf	0.486
Li_2HfN_2	-0.771	Li_3N, Hf	Li_3N, Hf	0.771
HfF_4	1.481	Li_2HfF_6, Hf	LiF, Hf	-1.173
Li_2HfF_6	1.019	Hf, LiF	LiF, Hf	-1.019
HfS_2	0.942	Li_2S, Hf_2S	Hf, Li_2S	-0.821

HfO_2	0.457	Hf, Li_2HfO_3	Hf, Li_2O	-0.077
$Li_6Hf_2O_7$	-0.153	Li_2O, Hf	Li_2O, Hf	0.153
Li_2HfO_3	0.122	$Li_6Hf_2O_7$, Hf	Li_2O, Hf	0.113
Zr_3N_4	0.956	Li_2ZrN_2, ZrN	Li_3N, Zr	0.374
Li_2ZrN_2	-0.603	Zr_2N, Li_3N	Zr, Li_3N	0.651
ZrF_4	1.619	$Li_3Zr_4F_{19}$, Zr	LiF, Zr	-1.331
Li_4ZrF_8	1.209	LiF, Zr	LiF, Zr	-1.209
Li_2ZrF_6	1.239	Li_4ZrF_8, Zr	LiF, Zr	-1.216
$Li_3Zr_4F_{19}$	1.510	Li_2ZrF_6, Zr	Zr, LiF	-1.277
ZrS_2	1.705	$Li(ZrS_2)_2$	Li_2S, Zr	-0.870
Li_2ZrS_3	1.227	$Li(ZrS_2)_2$, Li_2S	Zr, Li_2S	-0.810
ZrO_2	0.583	Li_2ZrO_3, Zr_3O	Zr, Li_2O	-0.232
$Li_6Zr_2O_7$	0.048	Li_2O, Zr_3O	Zr, Li_2O	-0.041
Li_2ZrO_3	0.339	$Li_6Zr_2O_7$, Zr_3O	Zr, Li_2O	-0.079
NaF	0.445	LiF, Na	Na, LiF	-0.445
Na_2S	0.417	Na, NaLiS	Na, Li_2S	-0.399
NaLiS	0.381	Na, Li_2S	Na, Li_2S	-0.381
Na_2O	0.925	Li_2O, Na	Na, Li_2O	-0.925
KF	0.449	LiF, K	LiF, K	-0.449
K_2S	0.488	KLiS, K	K, Li_2S	-0.410
KLiS	0.333	Li_2S, K	Li_2S, K	-0.333
K_2O	1.209	Li_2O, K	K, Li_2O	-1.209
Li_2CeN_2	-0.451	CeN, Li_3N	Ce, Li_3N	0.495
CeF_4	3.228	$LiCeF_5$, CeF_3	Ce, LiF	-1.257
$LiCeF_5$	3.158	CeF_3, LiF	Ce, LiF	-1.248
CeS_2	1.955	Ce_2S_3, Li_2S	Li_2S, Ce	-0.734
CeO_2	1.225	$Ce_{11}O_{20}$, Li_2CeO_3	Li_2O, Ce	-0.147
Li_2CeO_3	1.174	$Ce_{11}O_{20}$, Li_2O	Ce, Li_2O	-0.144
AlN	-0.004	Li_3AlN_2, Li_9Al_4	Li_3N, Li_9Al_4	0.160
Li_3AlN_2	-0.316	Li_3N, Li_9Al_4	Li_3N, Li_9Al_4	0.316
AlF_3	1.289	Li_3AlF_6, Al	Li_9Al_4, LiF	-0.767
Li_3AlF_6	1.057	Al, LiF	Li_9Al_4, LiF	-0.701
Al_2S_3	1.597	Al, $LiAlS_2$	Li_9Al_4, Li_2S	-0.740
$LiAlS_2$	0.973	Li_5AlS_4, Al	Li_9Al_4, Li_2S	-0.650
Li_5AlS_4	0.963	Al, Li_2S	Li_9Al_4, Li_2S	-0.647
Al_2O_3	1.233	$LiAl_5O_8$, Al	Li_9Al_4, Li_2O	-0.233
$LiAlO_2$	0.167	Li_5AlO_4, Li_3Al_2	Li_9Al_4, Li_2O	-0.105
Li_5AlO_4	0.056	Li_2O, Li_9Al_4	Li_9Al_4, Li_2O	-0.056
$LiAl_5O_8$	0.802	$LiAlO_2$, Al	Li_9Al_4, Li_2O	-0.195
Si_3N_4	1.127	$LiSi_2N_3$, Si	$Li_{21}Si_5$, Li_3N	-0.067
$LiSi_2N_3$	0.245	Li_2SiN_2, $Li_{13}Si_4$	Li_3N, $Li_{21}Si_5$	0.011
Li_2SiN_2	0.022	Li_5SiN_3, $Li_{21}Si_5$	Li_3N, $Li_{21}Si_5$	0.091
Li_5SiN_3	-0.148	Li_3N, $Li_{21}Si_5$	Li_3N, $Li_{21}Si_5$	0.148

SiF_4	2.298	Si, Li_2SiF_6	$Li_{21}Si_5$, LiF	-1.112
Li_2SiF_6	1.839	Si, LiF	LiF, $Li_{21}Si_5$	-1.038
SiS_2	1.633	Li_2SiS_3, Si	$Li_{21}Si_5$, Li_2S	-0.872
Li_2SiS_3	1.455	Li_8SiS_6, Si	$Li_{21}Si_5$, Li_2S	-0.840
Li_8SiS_6	1.411	Si, Li_2S	$Li_{21}Si_5$, Li_2S	-0.829
SiO_2	1.320	$Li_2Si_2O_5$, Si	$Li_{21}Si_5$, Li_2O	-0.454
Li_2SiO_3	0.761	Li_4SiO_4, Si	$Li_{21}Si_5$, Li_2O	-0.295
$Li_2Si_2O_5$	1.265	Li_2SiO_3, Si	$Li_{21}Si_5$, Li_2O	-0.372
Li_4SiO_4	0.258	$Li_{13}Si_4$, Li_8SiO_6	$Li_{21}Si_5$, Li_2O	-0.222
Li_8SiO_6	0.227	$Li_{13}Si_4$, Li_2O	Li_2O, $Li_{21}Si_5$	-0.213
Zn_3N_2	1.423	Zn, LiN_3	LiZn, Li_3N	-0.532
LiZnN	0.380	Li_3N, $LiZn_3$	LiZn, Li_3N	-0.365
ZnF_2	2.441	Zn, Li_2ZnF_4	LiZn, LiF	-1.764
Li_2ZnF_4	2.428	Zn, LiF	LiZn, LiF	-1.760
ZnS	1.200	Zn, Li_2S	LiZn, Li_2S	-0.941
ZnO	1.364	$Li_{10}Zn_4O_9$, Zn	Li_2O, LiZn	-1.014
Li_6ZnO_4	1.179	Li_2O, Zn	LiZn, Li_2O	-0.927
$Li_{10}Zn_4O_9$	1.318	Li_6ZnO_4, Zn	LiZn, Li_2O	-0.968
InN	1.037	LiN_3, In	Li_3N, $Li_{13}In_3$	-0.376
InF_3	2.625	In, $LiInF_4$	LiF, $Li_{13}In_3$	-1.195
$LiInF_4$	2.517	LiF, In	LiF, $Li_{13}In_3$	-1.184
In_2S_3	1.634	InS, Li_2S	$Li_{13}In_3$, Li_2S	-0.785
In_2O_3	1.579	$LiInO_2$, In	$Li_{13}In_3$, Li_2O	-0.734
$LiInO_2$	1.363	In, Li_2O	$Li_{13}In_3$, Li_2O	-0.712
EuN	-0.051	Li_3N, Eu	Li_3N, Eu	0.051
EuF_3	3.389	EuF_2, LiF	Eu, LiF	-1.278
Eu_2O_3	1.655	EuO, Li_2O	Li_2O, Eu	-0.531
Ta_3N_5	1.371	$Li_2Ta_3N_5$	Ta, Li_3N	0.073
Li_4TaN_3	-0.016	Li_7TaN_4, Ta	Li_3N, Ta	0.583
Li_7TaN_4	-0.771	Li_3N, Ta	Li_3N, Ta	0.771
$LiTaN_2$	0.877	$Li_2Ta_3N_5$, Li_4TaN_3	Li_3N, Ta	0.250
TaF_5	2.895	Ta, $LiTaF_6$	LiF, Ta	-2.045
$LiTaF_6$	1.876	LiF, Ta	LiF, Ta	-1.876
Li_3TaS_4	1.853	Li_2S, $Li_2(TaS_2)_3$	Li_2S, Ta	-1.302
Ta_2O_5	1.694	$LiTa_3O_8$, Ta	Li_2O, Ta	-0.758
$LiTaO_3$	1.175	Li_3TaO_4, Ta	Li_2O, Ta	-0.583
Li_3TaO_4	0.543	Li_5TaO_5, Ta	Ta, Li_2O	-0.386
$LiTa_3O_8$	1.591	$LiTaO_3$, Ta	Ta, Li_2O	-0.695
Li_5TaO_5	0.347	Ta, Li_2O	Li_2O, Ta	-0.347
Li_5TiN_3	-0.828	Li_3N, Ti_2N	Li_3N, Ti	0.829
Li_2TiN_2	0.712	TiN, Li_5TiN_3	Li_3N, Ti	0.572
TiF_4	2.928	TiF_3, Li_2TiF_6	Ti, LiF	-1.725
Li_2TiF_6	1.896	TiF_3, LiF	Ti, LiF	-1.553

TiS_2	1.939	$Li(TiS_2)_3$	Ti, Li_2S	-1.050
Li_2TiS_3	1.717	Li_2S, $LiTiS_2$	Ti, Li_2S	-1.009
TiO_2	1.712	$LiTi_2O_4$	Ti, Li_2O	-0.468
Li_2TiO_3	0.500	$LiTiO_2$, Li_4TiO_4	Ti, Li_2O	-0.167
Li_4TiO_4	0.123	Ti_3O, Li_2O	Ti, Li_2O	-0.095
$Li_4Ti_5O_{12}$	1.749	$Li_7Ti_{11}O_{24}$, Li_2TiO_3	Ti, Li_2O	-0.347
Li_2SnN_2	0.473	Li_3N, $Li_{13}Sn_5$	$Li_{17}Sn_4$, Li_3N	-0.424
SnF_4	4.319	SnF_3, Li_2SnF_6	LiF, $Li_{17}Sn_4$	-1.651
Li_2SnF_6	2.911	SnF_2, LiF	LiF, $Li_{17}Sn_4$	-1.528
SnS_2	1.945	Li_2SnS_3, SnS	Li_2S, $Li_{17}Sn_4$	-1.058
Li_2SnS_3	1.822	SnS, Li_2S	$Li_{17}Sn_4$, Li_2S	-1.043
SnO_2	1.986	Li_2SnO_3, Sn_5O_6	$Li_{17}Sn_4$, Li_2O	-0.955
Li_8SnO_6	1.243	Li_2O, Sn	$Li_{17}Sn_4$, Li_2O	-0.824
Li_2SnO_3	1.362	Li_8SnO_6, Sn	$Li_{17}Sn_4$, Li_2O	-0.853
BN	0.062	Li_3BN_2, LiB	Li_3N, LiB	0.195
Li_3BN_2	-0.453	Li_3N, LiB	LiB, Li_3N	0.453
BF_3	2.933	B, $LiBF_4$	LiF, LiB	-1.720
$LiBF_4$	1.937	B, LiF	LiB, LiF	-1.534
B_2S_3	1.870	B, $Li_5B_7S_{13}$	Li_2S, LiB	-1.319
Li_3BS_3	1.589	B, Li_2S	LiB, Li_2S	-1.272
$Li_5B_7S_{13}$	1.615	Li_3BS_3, B	Li_2S, LiB	-1.280
B_2O_3	2.067	B_6O, $Li_3B_7O_{12}$	LiB, Li_2O	-0.652
Li_3BO_3	0.284	$Li_7B_{18}O$, Li_2O	Li_2O, LiB	-0.249
$Li_2B_4O_7$	1.291	$LiBO_2$, B_6O	Li_2O, LiB	-0.508
$Li_3B_7O_{12}$	1.294	$Li_2B_4O_7$, B_6O	LiB, Li_2O	-0.519
$LiBO_2$	1.014	B_6O, Li_3BO_3	Li_2O, LiB	-0.436
GaN	0.484	Li_3GaN_2, LiGa	Li_2Ga, Li_3N	-0.281
Li_3GaN_2	0.125	Li_3N, Li_2Ga	Li_2Ga, Li_3N	-0.125
GaF_3	2.581	Li_3GaF_6, Ga	Li_2Ga, LiF	-1.637
Li_3GaF_6	2.277	Ga, LiF	Li_2Ga, LiF	-1.546
Ga_2S_3	2.099	$LiGaS_2$, GaS	Li_2Ga, Li_2S	-1.053
$LiGaS_2$	1.307	Ga, Li_2S	Li_2Ga, Li_2S	-0.964
Ga_2O_3	2.131	$LiGa_5O_8$, Ga	Li_2Ga, Li_2O	-0.902
$LiGaO_2$	1.046	Li_5GaO_4, Ga	Li_2Ga, Li_2O	-0.752
$LiGa_5O_8$	1.892	Ga, $LiGaO_2$	Li_2Ga, Li_2O	-0.865
Li_5GaO_4	0.862	Ga, Li_2O	Li_2Ga, Li_2O	-0.697
NiF_2	3.200	Ni, Li_2NiF_4	Ni, LiF	-3.165
Li_2NiF_4	3.130	Ni, LiF	Ni, LiF	-3.130
NiO	2.172	Li_2O, Ni	Li_2O, Ni	-2.172
Li_7NbN_4	-0.639	Li_3N, Nb	Nb, Li_3N	0.639
$LiNbN_2$	0.787	NbN, Li_7NbN_4	Li_3N, Nb	0.092
NbF_5	3.169	Nb_2F_5, $LiNbF_6$	Nb, LiF	-2.309
$LiNbF_6$	2.204	Nb_2F_5, LiF	Nb, LiF	-2.171

Li_3NbS_4	2.083	Li_2S, $Li_5(NbS_2)_7$	Nb, Li_2S	-1.378
Nb_2O_5	2.286	$LiNb_3O_8$, $Nb_{12}O_{29}$	Li_2O, Nb	-0.970
$LiNbO_3$	1.745	Li_3NbO_4, $LiNbO_2$	Li_2O, Nb	-0.814
$LiNb_3O_8$	2.225	$Nb_{12}O_{29}$, $LiNbO_3$	Nb, Li_2O	-0.917
$Li_8Nb_2O_9$	0.623	$LiNbO_2$, Li_2O	Nb, Li_2O	-0.577
Li_3NbO_4	0.937	$Li_8Nb_2O_9$, $LiNbO_2$	Nb, Li_2O	-0.603
FeN	0.630	Fe_3N, Li_3FeN_2	Li_3N, Fe	-0.405
Li_3FeN_2	0.188	Li_3N, Fe	Fe, Li_3N	-0.188
FeF_3	3.655	$LiFe_2F_6$	Fe, LiF	-2.663
Li_3FeF_6	3.151	FeF_2, LiF	Fe, LiF	-2.553
Fe_2O_3	2.299	Fe_3O_4, $LiFeO_2$	Fe, Li_2O	-1.535
Li_5FeO_4	1.282	Li_2O, Fe	Fe, Li_2O	-1.282
$LiFeO_2$	1.540	Li_2FeO_2	Fe, Li_2O	-1.373
Ge_3N_4	1.728	$LiGe_2N_3$, Ge	Li_3N, $Li_{15}Ge_4$	-0.438
Li_5GeN_3	0.198	$Li_{15}Ge_4$, Li_3N	$Li_{15}Ge_4$, Li_3N	-0.198
$LiGe_2N_3$	0.735	Li_2GeN_2, Ge	Li_3N, $Li_{15}Ge_4$	-0.356
Li_2GeN_2	0.500	Li_5GeN_3, LiGe	$Li_{15}Ge_4$, Li_3N	-0.282
GeF_4	3.773	Ge_5F_{12}, Li_2GeF_6	LiF, $Li_{15}Ge_4$	-1.735
Li_2GeF_6	2.710	LiF, Ge	$Li_{15}Ge_4$, LiF	-1.598
GeS_2	1.913	GeS, Li_2GeS_3	Li_2S, $Li_{15}Ge_4$	-1.107
Li_4GeS_4	1.622	Li_2S, Ge	Li_2S, $Li_{15}Ge_4$	-1.037
Li_2GeS_3	1.865	Li_4GeS_4, Ge	$Li_{15}Ge_4$, Li_2S	-1.068
GeO_2	2.408	$Li_2Ge_7O_{15}$, Ge	$Li_{15}Ge_4$, Li_2O	-0.992
Li_4GeO_4	1.016	Ge, Li_2O	Li_2O, $Li_{15}Ge_4$	-0.724
$Li_2Ge_7O_{15}$	2.275	$Li_4Ge_5O_{12}$, Ge	Li_2O, $Li_{15}Ge_4$	-0.960
$Li_4Ge_5O_{12}$	2.259	Li_2GeO_3, Ge	Li_2O, $Li_{15}Ge_4$	-0.911
Li_2GeO_3	1.579	Ge, Li_4GeO_4	Li_2O, $Li_{15}Ge_4$	-0.797
CuF_2	3.638	LiF, Cu	$LiCu_3$, LiF	-3.136
CuS	1.821	Cu_7S_4, Li_2S	$LiCu_3$, Li_2S	-1.569
CuO	2.411	LiCuO	$LiCu_3$, Li_2O	-1.863
WN_2	2.056	$Li_5W_7N_{12}$, N_2	Li_3N, W	-0.353
Li_6WN_4	-0.465	Li_3N, W	Li_3N, W	0.465
WF_6	3.593	$LiWF_6$	W, LiF	-3.067
WO_3	2.564	Li_2WO_4, $W_{18}O_{49}$	W, Li_2O	-1.650
Li_2WO_4	2.097	Li_4WO_5, W	Li_2O, W	-1.394
Li_4WO_5	1.219	W, Li_2O	W, Li_2O	-1.219
YbS_2	2.154	YbS, LiS_4	Yb, Li_2S	-0.947
YbO_2	2.853	YbO, Li_2O	Yb, Li_2O	-1.155
P_3N_5	2.286	$LiPN_2$, P	Li_3P, Li_3N	-0.552
$LiPN_2$	0.610	Li_7PN_4, Li_3P	Li_3P, Li_3N	-0.307
Li_7PN_4	0.005	Li_3P, Li_3N	Li_3P, Li_3N	-0.005
PF_5	3.302	P, $LiPF_6$	Li_3P, LiF	-2.118
$LiPF_6$	2.735	P, LiF	Li_3P, LiF	-2.059

P_2S_5	2.273	Li_3PS_4, P_4S_9	Li_3P, Li_2S	-1.532
Li_3PS_4	1.719	P, Li_2S	Li_3P, Li_2S	-1.423
P_2O_5	3.274	$LiPO_3$, P	Li_3P, Li_2O	-1.215
$Li_4P_2O_7$	2.307	Li_3PO_4, P	Li_3P, Li_2O	-0.826
Li_3PO_4	0.688	Li_2O, Li_3P	Li_3P, Li_2O	-0.688
$LiPO_3$	2.479	P, $Li_4P_2O_7$	Li_3P, Li_2O	-0.979
Li_6MoN_4	-0.362	Li_3N, Mo	Li_3N, Mo	0.362
MoF_6	4.335	Li_2MoF_6	LiF, Mo	-3.257
MoO_3	3.350	Mo_8O_{23}, Li_2MoO_4	Mo, Li_2O	-1.757
Li_2MoO_4	1.842	Li_2MoO_3, Li_4MoO_5	Li_2O, Mo	-1.420
Li_4MoO_5	1.333	Mo, Li_2O	Mo, Li_2O	-1.333
Li_7VN_4	-0.718	Li_3N, V	Li_3N, V	0.718
$LiVN_2$	0.841	VN, Li_7VN_4	Li_3N, V	0.083
VF_5	5.104	VF_4, $LiVF_6$	V, LiF	-2.968
$LiVF_6$	4.570	Li_2VF_6	V, LiF	-2.849
Li_3VS_4	1.795	V_3S_4, Li_2S	V, Li_2S	-1.433
V_2O_5	3.675	$LiVO_3$, V_3O_7	V, Li_2O	-1.493
Li_3VO_4	1.126	Li_2O, $LiVO_2$	Li_2O, V	-0.947
$LiVO_3$	3.229	LiV_2O_5, Li_3VO_4	V, Li_2O	-1.287
Li_2MnN_2	0.552	Li_7MnN_4, Mn_2N	Li_3N, Mn	-0.092
MnF_4	6.030	MnF_3, Li_2MnF_6	LiF, Mn	-3.353
Li_2MnF_6	4.557	LiF, Li_2MnF_5	LiF, Mn	-3.065
MnS_2	1.830	$Li(MnS_2)_2$	Mn, Li_2S	-1.598
MnO_2	3.711	Mn_2O_3, $Li_5Mn_7O_{16}$	Li_2O, Mn	-1.748
Li_2MnO_3	1.732	Li_2O, $LiMnO_2$	Mn, Li_2O	-1.381
$Li_4Mn_5O_{12}$	3.417	$Li_5Mn_7O_{16}$, Li_2MnO_3	Mn, Li_2O	-1.565
CoF_4	6.635	$LiCoF_4$	LiF, Co	-4.150
CoS_2	1.984	Co_3S_4, Li_2S	Co, Li_2S	-1.750
CoO_2	4.096	$Li(CoO_2)_2$	Co, Li_2O	-2.253
Li_8CoO_6	2.605	Li_5CoO_4, Li_2O	Co, Li_2O	-1.943

3. Comparison between DFT calculated and experimental values

To evaluate the error of the DFT calculation results, we compared the formation enthalpy of binary nitrides and the potential (referenced to Li/Li^{+}) of lithium conversion reaction of binary nitrides based on the following reaction

$$M_xN_y + 3y\ Li \rightarrow y\ Li_3N + x\ M.$$

These results are summarized and compared in Table S5. We found that the calculated conversion reaction potential is general in agreement with the experimental value with an error of less than 0.1 V.

Table S5. Experimental and calculated formation enthalpy and conversion reaction potential of binary nitrides.

	$\Delta H_{exp.}$ * (eV/atom)	$\Delta H_{cal.}$ (eV/atom)	$\phi_{exp.}$ (V)	$\phi_{cal.}$ (V)
Li_3N	-0.428	-0.460	-	-
TaN	-1.309	-1.366	-0.303	-0.297
Ca_3N_2	-0.912	-0.946	-0.190	-0.175
GaN	-0.569	-0.666	0.191	0.169
VN	-1.131	-1.164	-0.184	-0.162
NbN	-1.227	-1.252	-0.248	-0.221
Be_3N_2	-1.224	-1.233	-0.450	-0.414
HfN	-1.939	-1.940	-0.722	-0.680
Cr_2N	-0.434	-0.523	0.136	0.091
CeN	-1.694	-1.684	-0.559	-0.509
Ta_2N	-0.942	-0.934	-0.372	-0.321
Zn_3N_2	-0.047	-0.033	0.531	0.586
TiN	-1.754	-1.906	-0.600	-0.657
UN	-1.529	-1.501	-0.449	-0.387
BN	-1.309	-1.472	-0.303	-0.368
LaN	-1.553	-1.517	-0.465	-0.398
ZrN	-1.911	-1.870	-0.704	-0.633
Si_3N_4	-1.104	-1.312	-0.074	-0.152
AlN	-1.652	-1.595	-0.531	-0.450
Mg_3N_2	-0.958	-0.909	-0.228	-0.144
CrN	-0.608	-0.527	0.165	0.262
Th_3N_4	-1.935	-1.829	-0.559	-0.453
PuN	-1.553	-1.438	-0.465	-0.345
EuN	-1.129	-0.996	-0.183	-0.051
YN	-1.553	-1.879	-0.465	-0.639
ThN	-1.965	-1.761	-0.740	-0.561
ScN	-1.628	-2.106	-0.515	-0.790
InN	-0.717	-0.105	0.092	0.543

* Chase, M. W. NIST-JANAF thermochemical tables 4th ed.; National Institute of Standards and Technology: Gaithersburg, 1998.

4. Li-M-O-N grand potential phase diagrams

Figure S1. Grand potential phase diagrams in equilibrium with Li metal. The composition ranges that form passivating interphase and that form non-passivating interphase are colored green and red, respectively.

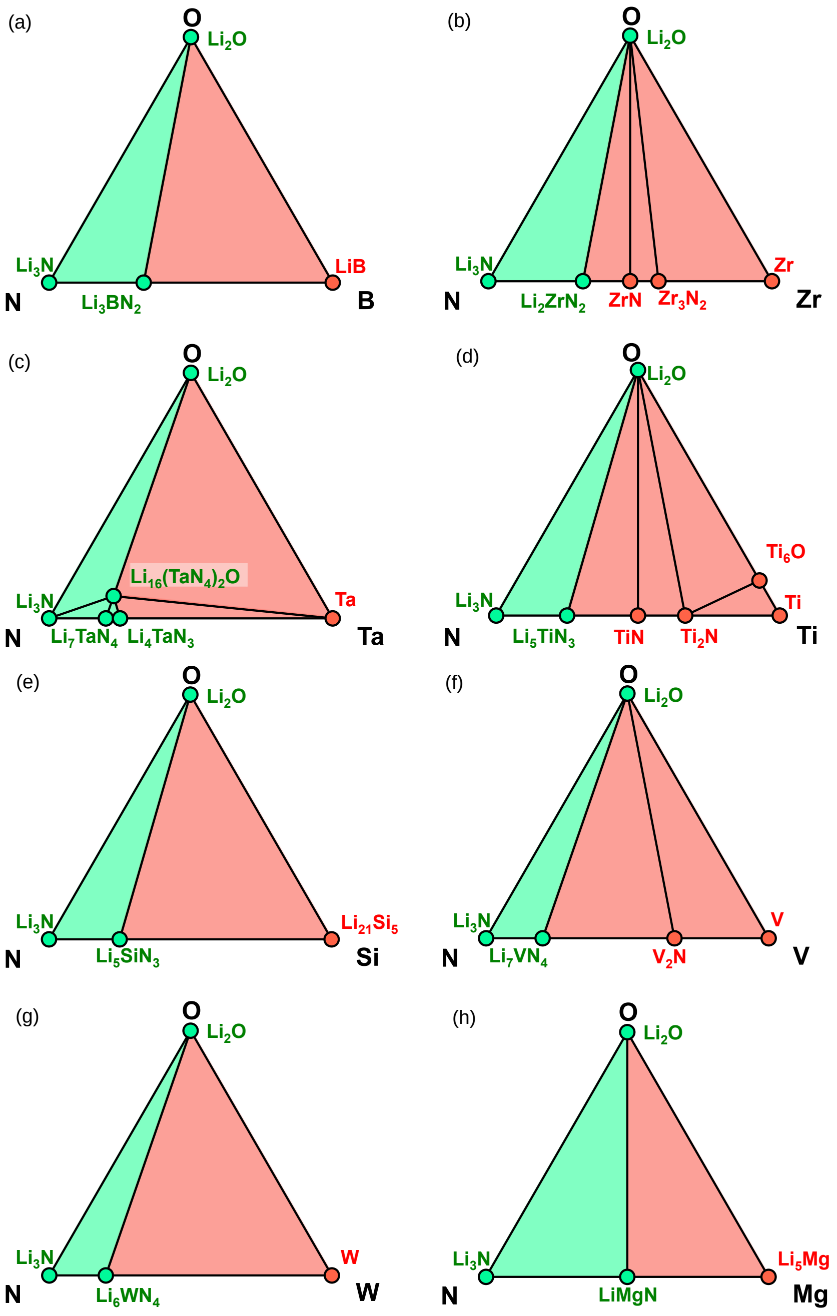

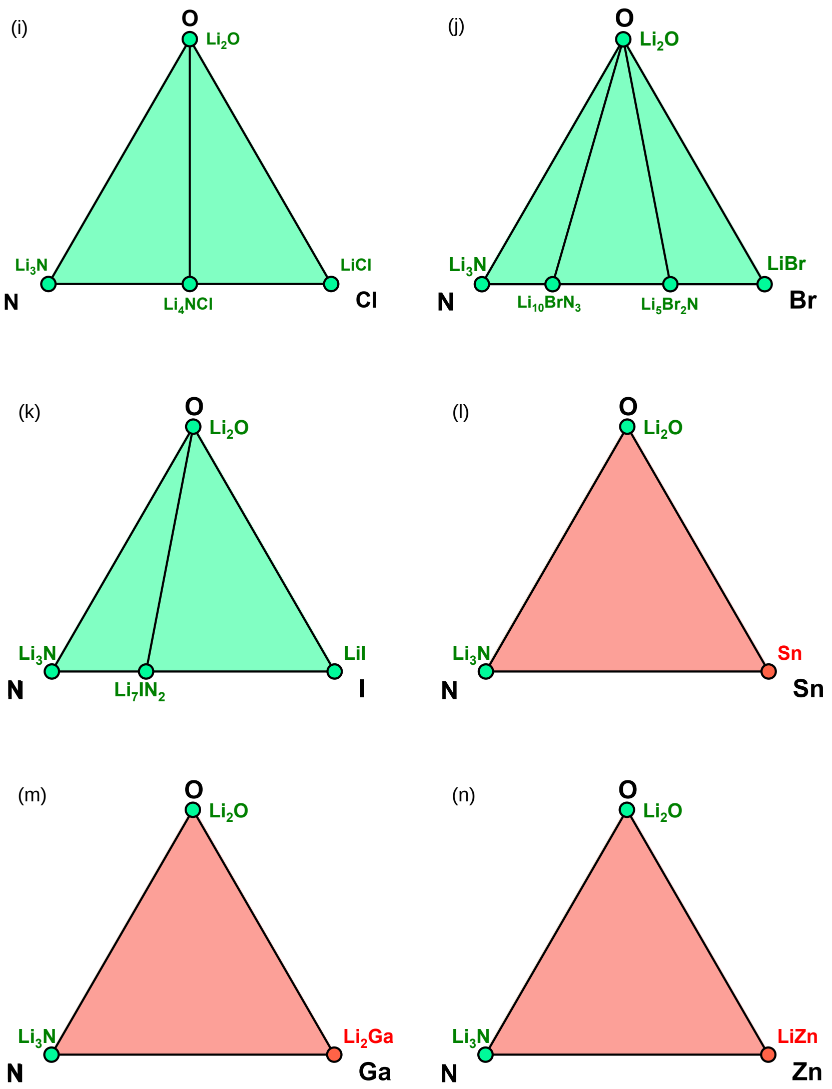
(i)
O
Li_2O
Li_3N
LiCl
N
Li_4NCl
Cl
(j)
O
Li_2O
Li_3N
LiBr
N
$Li_{10}BrN_3$
Li_5Br_2N
Br
(k)
O
Li_2O
Li_3N
LiI
N
Li_7IN_2
I
(l)
O
Li_2O
Li_3N
Sn
N
Sn
(m)
O
Li_2O
Li_3N
Li_2Ga
N
Ga
(n)
O
Li_2O
Li_3N
LiZn
N
Zn
